# Surgery

**Published:** 1898-09

**Authors:** W. H. Harsant


### SURGERY.

It has long been the aim of surgeons to devise a means by which the interior of the **male bladder may be illuminated** and seen in the same manner and with the same safety that the vocal cords in the larynx or the fundus of the eye may be examined. For this purpose various kinds of cystoscope have been introduced at different times; but all were more or less unsatisfactory until Dr. Max Nitze and Mr. Leiter together worked out the details of an electric cystoscope, by which a small electric lamp might be introduced into the bladder so as to light up its interior while an examination of its walls might be made by a special optical apparatus placed in the interior of the shaft. At first the instrument was encumbered by an elaborate water cooling apparatus, for preventing the elaboration of undue heat which might injure the bladder walls: but at a later period various improvements were introduced by which this cooling apparatus might be dispensed with; and since Hurry Fenwick[1] devoted much time and trouble to perfecting the electric cystoscope we have been possessed of an instrument which *in skilful hands*, and when used with all due precautions, has served a very useful purpose and has undoubtedly been of much service. But at the same time there were many objections to it, some of them of so serious a nature as to prevent its very frequent use. Among them we may notice the following: (1) Considerable practice is necessary for its successful manipulation. In the hands of Hurry Fenwick and others, who have long worked with it, it may be made to yield valuable results; but when used by the average surgeon, with only scant opportunities for practice, the result is often most disappointing. (2) It is necessary to have five or six ounces of a perfectly clear fluid in the bladder. In cases of tumour of the bladder with free hemorrhage this is frequently impossible, and hence its use is often contra-indicated when it is most desired. (3) Numerous accidents have occurred from its use, especially from burning the walls of the bladder and from breakage of the lamps.

An immense advance in cystoscopy has, however, lately been made by Dr. Howard Kelly.[2] When he first succeeded in his vesical and ureteral examinations in women in the spring of 1893, he expressed a conviction that the bladder in the male could also be investigated in a similar manner. So positive was his conviction that he had a long, straight, male cystoscope made by Messrs. F. Arnold & Sons, of Baltimore, on November 18th, 1893. He had no opportunity for testing this instrument until November, 1897, when he was able to try it on a man of about forty-eight years old, who had a persistent hæmaturia of undetermined origin. After due antiseptic precautions and washing out the bladder, it was filled with a saline solution and first examined with the ordinary cystoscope. For the most part the bladder-wall was found normal; but at the base a dark, tufted, villous area was found from which a cloud of blood kept rising and mingling with the clear medium. The conclusion was reached that this was probably a vesical papilloma. The bladder was then emptied with a catheter, and the patient

[1] *The Electric Illumination of the Bladder and Urethra*, by E. Hurry Fenwick, 2nd Ed., 1889.

[2] *Ann. Surg.*, 1898, xxvii. 71, 475.

placed in the knee-breast position, with the chest close down to the table, the elbows spread apart, and the thighs slightly drawn up under the abdomen. The straight cystoscope was then introduced, eight millimetres in diameter, and with a tube eighteen centimetres long, with a funnel-shaped opening and a diminutive handle like those attached to his first cystoscopes used in women.

This cystoscope was inserted into the bladder without difficulty, while the patient was still in the dorsal position ; when he assumed the knee-breast position the obturator was drawn out, and air at once entered the bladder. The examination of the bladder was now conducted by looking into it through an ordinary head-mirror, reflecting an electric light which was held close to the sacrum. The base of the bladder came perfectly into view, and the posterior walls showed the characteristic pallor, with small vessels branching here and there over its surface ; it could be seen that the base was simply coated with blood which had accumulated there in the most dependent position, and that there was no papilloma or other growth present. The trigonum and the interureteric ligament were injected and plainly defined.

Later experiments by Dr. Kelly demonstrated that the first speculum was longer than necessary, and that its calibre might be made larger without risk of injuring the average urethra. The cystoscope he now uses consists of an open cylindrical tube, eight millimetres in diameter, fifteen and a half centimetres long, with a funnel-shaped opening twenty millimetres long and twenty-seven millimetres in diameter at its outer orifice, blackened on the inside and on the rim to avoid the reflection of the light. A stout handle, eleven centimetres long and twenty-five by twelve millimetres thick, is attached to the funnel, and affords a good grasp to the hand, enabling it to control the speculum perfectly. The instrument is best introduced while the patient lies on his back, and when it has entered the bladder the patient is aided in rolling over on his side, and then his face, and then in getting up on his hands and knees. This is managed by the assistants, while the surgeon is engaged in retaining the speculum in place.

In taking the knee-breast position it is important to see that he brings his chest close down to the table with the elbows spread out wide apart and the face turned sideways, at the same time the thighs are vertical or slightly drawn up under the abdomen. If he is allowed to crouch or keep his back arched, or to take any other of the numerous positions patients try to assume than the one just described, the examination will probably prove unsatisfactory, as the bladder will not distend with air.

The next step is to introduce a small speculum into the rectum, to allow it to fill with air : if this is not done, the bladder expands so much that the base of it disappears up in the direction of the sacral hollow out of sight; by distending the rectum with air the base of the bladder drops down into the plane of vision and within easy reach of inspection. The obturator is now withdrawn from the cystoscope, when the atmospheric air enters and distends the bladder with an audible suction sound.

The illumination of the interior of the bladder is effected either by direct light, which is the best way, or by a light held close to the sacrum and reflected by a head-mirror of about twenty-five cubic centimetres focal length. If an electric light, sixteen candle power, it is held close to the sacrum, with a white enamel-painted tin reflector back of it; an ordinary head-mirror will direct sufficient light into the interior of the bladder to enable one who is practised in this way of examining patients to see all the details with perfect clearness.

Dr. Weir pointed out to Dr. Kelly that the use of straight tubes for inspecting a limited portion of bladder, with the patient in the dorsal position, dates as far back as the work of Desormeaux, published in 1865. The procedure, from being one of a very limited utility, has been elevated into an important method by the postural atmospheric distension of the bladder; one which bids fair to largely supplant the electro-cystoscopic methods of Nitze, both in the fields of diagnosis and treatment.

W. H. Harsant.

